# Lipid Membranes as Key Targets for the Pharmacological Actions of Ginsenosides

**DOI:** 10.3389/fphar.2020.576887

**Published:** 2020-09-11

**Authors:** Sandrine L. Verstraeten, Joseph H. Lorent, Marie-Paule Mingeot-Leclercq

**Affiliations:** ^1^ Cellular & Molecular Pharmacology Unit (FACM), Louvain Drug Research Institute (LDRI), Université Catholique de Louvain (UCL), Brussels, Belgium; ^2^ Membrane Biochemistry & Biophysics, Bijvoet Center for Biomolecular Research, Utrecht University, Utrecht, Netherlands

**Keywords:** ginsenosides, biophysical membrane properties, lipid dynamics and membrane organization, anticancer, anti-infectious agents

## Abstract

In this review, we will focus on the activity of ginsenosides on membranes and their related effects, from physicochemical, biophysical, and pharmacological viewpoints. Ginsenosides are a class of saponins with a large structural diversity and a wide range of pharmacological effects. These effects can at least partly be related to their activity on membranes which results from their amphiphilic character. Some ginsenosides are able to interact with membrane lipids and associate into nanostructures, making them possible adjuvants for vaccines. They are able to modulate membrane biophysical properties such as membrane fluidity, permeability or the formation of lateral domains with some degree of specificity towards certain cell types such as bacteria, fungi, or cancer cells. In addition, they have shown antioxidant properties which protect membranes from lipid oxidation. They further displayed some activity on membrane proteins either through direct or indirect interaction. We investigate the structure activity relationship of ginsenosides on membranes and discuss the implications and potential use as anticancer, antibacterial, and antifungal agents.

## Introduction

The plasma membrane is the interface between the inter- and intracellular spaces. A major function of the plasma membrane is to transmit signals from the exterior to the interior of the cell. This makes it a rational target for pharmacological agents that modulate signaling and subsequently provoke a physiological or cellular response (Lorent et al., 2013). The majority of drugs act on membrane proteins (Lorent et al., 2017); however, lipids are also important for signal transduction. Lipids can collectively form signaling platforms or lateral domains, which include or exclude certain types of proteins. Analysis of the plasma membrane lipidome has highlighted a great variety of membrane lipids (van Meer, 2005). However, it remains unknown whether each individual lipid has its own function or whether, collectively, lipids confer biophysical properties to the membrane, and hence modulate cellular functions.

Among the active ingredients of ginseng are saponins, most of which are glycosides of triterpenoid aglycones (Shin et al., 2015). Ginseng saponins, also called ginsenosides, are numerous with a high chemical variation, depending on the linkage position and numbers of sugars on the aglycone skeleton (He et al., 2018). Ginsenosides are membrane-active substances, which modulate membrane dynamics and lateral organization in domains (Kwon et al., 2008; Park et al., 2010; Lorent J. et al., 2014). Interestingly, the mechanism of action of most ginsenosides does not seem to involve the formation of pores or holes in the membrane, as observed for other saponins such as digitonin (Lorent J. H. et al., 2014) or α-hederin (Lorent et al., 2013), reducing the risk of hemolysis. The traditional use of ginseng suggests that ginsenosides are relatively safe *in vivo* and constitute interesting pharmacological agents. In this review, we will focus on the activity of ginsenosides on membranes and their related effects, from physicochemical, biophysical, and pharmacological viewpoints (**Table 1**).

**Table 1:** Interaction of ginsenosides with membranes and pharmacological consequences.

Direct effects of ginsenoside(s)	Ginsenoside(s)	Method	Model	Pharmacological consequence(s)	References
Membrane dynamics					
Compacts the hydrophobic membrane core	Rh2	Fluorescence anisotropy of DPH	B16 melanoma cells	Induces flattening, increases adhesiveness to plastic surfaces, agglutinability of B16 cells	(Ota et al., 1987)
Relaxes the interfacial packaging of the polar head of PL	Rh2	Fluorescence anisotropy of DPH	U937 leukemia cells	Inactivates Akt and induces cancer cell apoptosis	(Verstraeten et al., 2018)
Compacts the hydrophobic membrane core	Rh2	Fluorescence anisotropy of DPH and TMA-DPH	B16 melanoma cells	Induces dendrite formation in melanoma cells	(Jiang et al., 2010)
Increases membrane fluidity	Rb2, Rc, Rd Re, Rf, Rg1, Rg2, Rh2, PPD	Two-photon fluorescence microscopy with carboxy-laurdan	HeLa cervical carcinoma cells	Activates death receptors and induces cancer cells apoptosis	(Yi et al., 2009)
Compacts the hydrophobic membrane core	Rh1	Fluorescence anisotropy of DPH	B16 melanoma cells	Does not induce changes in cell morphology and exerts no effect on cell adhesiveness	(Ota et al., 1987)
Compacts the hydrophobic membrane core	Rg3	Fluorescence anisotropy of DPH	Bovine chromaffin cells	Inhibits catecholamine secretion from cells stimulated by acetylcholine	(Tachikawa et al., 2001)
Compacts the hydrophobic membrane core and the interfacial packaging	Rg3	Fluorescence anisotropy of DPH and TMA-DPH	KB V20C (resistant) and parental KB (sensitive) cells	Inhibits efflux pumps in KB V20C	(Kwon et al., 2008)
Relaxes the hydrophobic membrane core	Re	Fluorescence anisotropy of DPH	Brain mitochondrial membrane from male Wistar rats	Protects rat brain against cerebral ischemia/reperfusion injury	(Zhou et al., 2006)
Relaxes the hydrophobic membrane core	Rg1	Fluorescence anisotropy of DPH	Old cortical cells from Wistar rats	Exerts anti-aging action	(Li and Zhang, 1997)
Relaxes the hydrophobic membrane core	Korean red ginseng (Rb1, Rg1, Re, Rb2)	Fluorescence anisotropy of DPH	*Candida Albicans*	Disrupts the fungal membrane	(Sung and Lee, 2008a)
Reduces the segmental chain mobility of the spin-labeled eggPC	Rc	Electron spin resonance	MultiLamellar Vesicles (MLVs) containing eggPC	Exhibits agglutinability toward eggPC vesicles	(Fukuda et al., 1987)
Reduces the segmental mobility of the spin-labeled eggPC	Rb2	Electron spin resonance	MLVs containing eggPC	No agglutinability toward eggPC vesicles	(Fukuda et al., 1987)
**Interaction with rafts**					
Reorganizes lipid rafts	Rp1	Detergent-Resistant membrane (DRM), immunofluorescence GM-1 staining	OVCAR-8 (sensitive), NCI/ADR-RES cells (resistant)	Redistributes raft-associated MDR-1 protein, enhances doxorubicin accumulation in drug-resistant cells	(Yun et al., 2013)
Disrupts the integrity of lipid rafts	Rg3	DRM, immunocytochemistry flotillin-1 staining	CHO cells, mouse primary neurons, brains from a mouse model of Alzheimer’s disease	Reduces the association of presenilin 1 (PS1) fragments with lipid rafts, inhibits λ-secretase activity Decreases amyloid-β (Aβ) levels	(Kang et al., 2013)
Reduces cholesterol and lipid raft levels	Rh2	Amplex Red Cholesterol Assay Kit, immunofluorescence GM-1 staining	Mouse primary neurons	Reduces Aβ secretion, amyloid precursor protein (APP) endocytosis, improves learning and memory function in Alzheimer’s disease	(Qiu et al., 2014)
Reduces lipid rafts and caveolae levels, increases their internalization	Rh2	Immunofluorescence GM-1 and caveolin-1 staining, DRM	A431, MBA-MB-231, PC3, HEK293 cells	Inactivates raft-associated Akt signaling and induces cancer cell apoptosis	(Park et al., 2010)
Disrupts the integrity of lipid rafts	Rb2, Rc, Rd Re, Rf, Rg1, Rg2, Rh2, PPD	Detergent-resistant membrane, immunofluorescence caveolae staining	HeLa cervical carcinoma cells	Activates death receptors and induces cancer cell apoptosis	(Kang et al., 2013)
Disrupts the integrity of lipid rafts	PPD	Immunofluorescence SM and CHOL staining, western blot of lipid raft-associated proteins (IGF-1R, P-Akt)	K562, HT29 cells, K562-xenografted BALB/c nude mice	Activates neutral SMase 2 and induces cancer cell apoptosis, reduces tumor volumes in xenograft mouse models	(Park et al., 2013)
**Membrane permeabilization and pore formation**					
Disturbs osmotic behavior of liposomes with or without Chol	Rb1	Absorbance measurements	MLVs composed of eggPC, PA with or without Chol	Induces liposomal membrane permeability	(Yu et al., 1985)
Disrupts the fungal membrane	Korean red ginseng (Rb1, Rg1, Re, Rb2)	Colony forming units (CFUs), fluorescence anisotropy of DPH	*Candida albicans*	Decreases membrane potential and exerts antifungal effects	(Sung and Lee, 2008a)
Disrupts the bacterial membrane	Korean red ginseng (Rb1, Rg1, Re, Rb2)	Calcein release in liposome, colony forming units	Liposome composed of PC/PG (1:1), *Staphylococcus aureus, S. epidermidis, Salmonella typhimurium*	Enhances kanamycin activity and exerts antibacterial effects	(Sung and Lee, 2008a)
Forms membrane pore	Rh2, Rg3	Absorbance of the supernatant	Red blood cells	Induces hemolysis	(Li and Liu, 2008)
Permeabilizes the lysosomal membrane	Octyl ester Rh2 derivative	Acridine orange relocation	HepG2 liver cells	Releases cathepsin from lysosomes to the cytosol compartment, induces cancer cell apoptosis	(Chen et al., 2016)
Disrupts the fungal membrane	Rg2, Rg3, Rg6, F4, Rg5, Rk1	Minimal fungicidal concentration (MFC)	*Epidermophyton floccosum, Trichophyton rubrum, T. mentagrophytes*	Decreases membrane potential and exhibit antifungal effects	(Xue et al., 2017)
Disrupts the bacterial membrane	Rg2, Rg3, Rg6, F4, Rg5, Rk1	Minimal bactericidal concentration (MBC), cell constituents released into cell suspension	*Fusobacterium nucleatum, Clostridium perfringens, Porphyromonas gingivalis*	Decreases the membrane potential and exerts antibacterial effects	(Xue et al., 2017)
**Interaction with membrane proteins**					
Decreases the activity of raft-associated Akt	PPD	DRM	U87 MG glioma cells	Enhances the chemotoxicity of paclitaxel or vinblastine	(Liu et al., 2011)
Increases the activity of raft-associated Akt	PPD	DRM	N2a neuroblastoma cells	Attenuates the excitotoxicity of N-methyl-D- aspartate	(Liu et al., 2011)
Interacts with the 5-HT3A receptor and the human Kv1.4 channel	Rg3	Ligand-gated ion currents measured *via* two-electrode voltage clamp technique, site-directed mutagenesis	*Xenopus laevis* oocytes	Inhibits 5-HT3A receptor-mediated ion currents and K^+^ currents flowing through the human Kv1.4 channel	(Lee et al., 2004; Lee et al., 2008)
Interacts with the NMDA receptor	Rg3, Rh2	Ligand-gated ion currents measured *via* two-electrode voltage clamp technique	Cultured rat hippocampal neurons	Antagonizes NMDA receptors	(Kim et al., 2004; Lee et al., 2006)
Interacts with the NorA efflux pump	Rh2	*In silico* molecular docking, rhodamine 123 retention assay	*S. aureus* *in vivo, S. aureus-*infected peritonitis mice	Promotes ciprofloxacin accumulation	(Zhang et al., 2014)
Interacts with Na^+^/K^+^ ATPase	PPD, Rh2, Rg3	Measurement of inorganic phosphate liberated from ATP and molecular modeling and docking	Na^+^/K^+^-ATPase from the porcine cerebral cortex	Inhibits Na^+^/K^+^ ATPase activity, exerts cardiac therapeutic effects	(Chen et al., 2009)
Binds to the P-gp efflux pump	Rg3	Rhodamine 123 retention assay and competition assay with [3H]azidopinen for binding to P-gp	Multidrug-resistant P388/DOX cells	Blocks drug efflux and enhances anticancer drug accumulation	(Kim et al., 2003)
**ROS production**					
Attenuates lipid peroxidation	Re	Antioxidant enzymes and measurement of reactive oxygen species	Ischemic brain tissues of male Wistar rats	Protects rat brain against cerebral ischemia/reperfusion injury	(Zhou et al., 2006)
Inhibits mitochondrial permeability transition by free radical scavenging action	Rg3	ROS measurements	Isolated rat brain mitochondria	Exerts a neuroprotective effect after cerebral ischemia	(Tian et al., 2009)
Inhibits hydroxyl radical formation	Rd	ROS measurements	Rat model of focal cerebral ischemia	Exerts neuroprotection in transient focal ischemia	(Ye et al., 2011a)
Inhibits lipid peroxidation	Rb1, Rg1	ROS measurements	Rat liver and brain microsomes	Prevents cardiac ischemia	(Deng and Zhang, 1991)
Exerts anti-oxidative effects	Rc > Rb1 and Re > Rd > R1 > Rg1 > Rb3 > Rh1	Measurement of the absorbance of erythrocyte supernatant	2-amidinopropane hydrochloride -induced hemolysis of erythrocytes	Reduces hemolysis	(Liu et al., 2003)
Exerts pro-oxidative effects	Rh2, Rg3	ROS measurements	Jurkat leukemia cells	Induces cancer cell apoptosis	(Xia et al., 2017)
Exerts pro-oxidative effects	Rh4	ROS measurements	Human colorectal cancer xenograft mouse model	Inhibits tumor growth	(Wu et al., 2018)

## Structural Diversity of Ginsenosides

**Figure 1:**
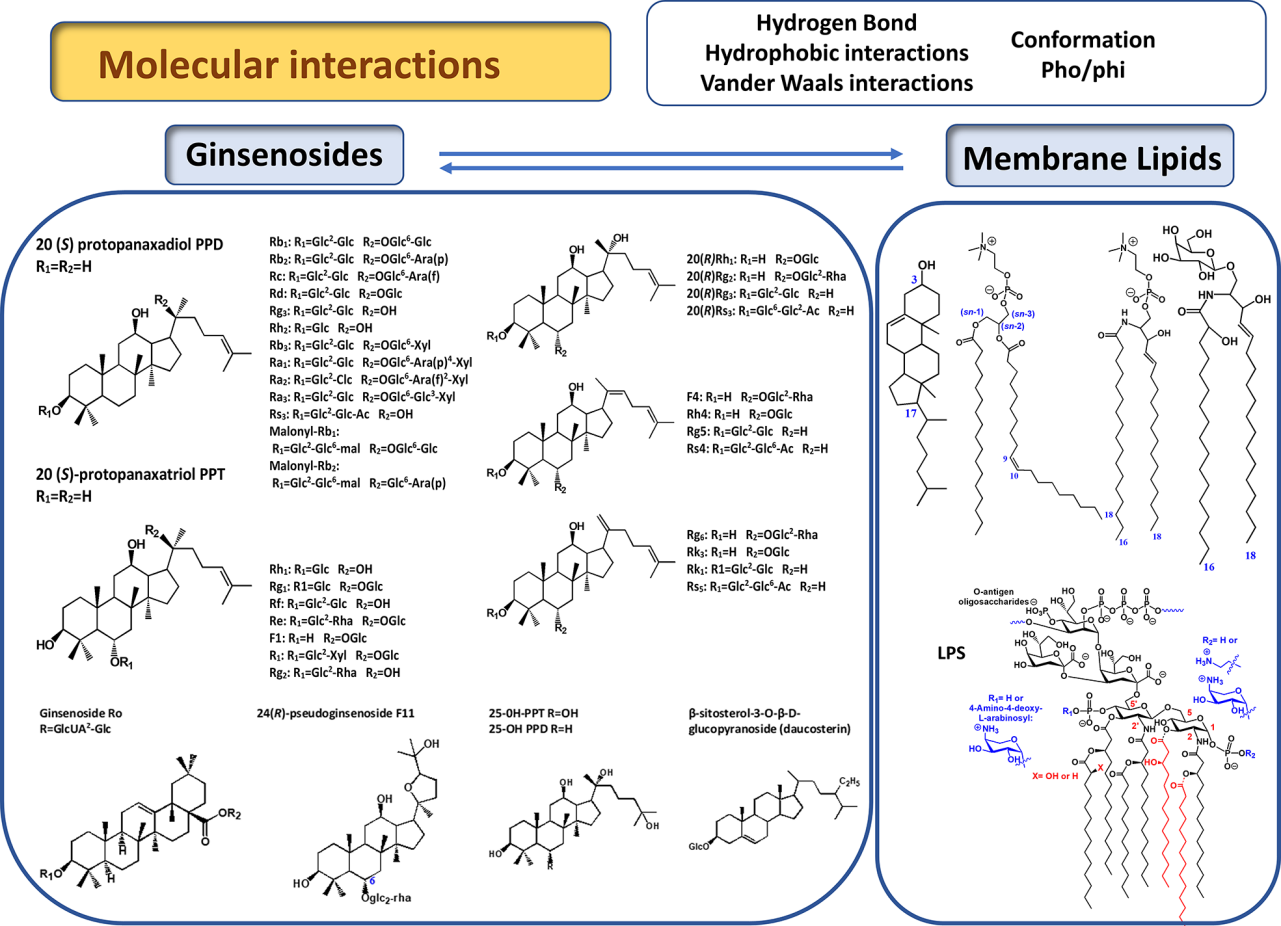
Chemical structure of main ginsenosides discussed in this review (Qi et al., 2011). Represented lipids are, from left to right, glycerophospholipids (phosphatidylcholine), sphingolipids (sphingomyelin, glycosphingolipids), sterols (cholesterol).

The structural diversity of ginsenosides is mainly a consequence of the high variety of sugar chains connected to different aglycone backbones. Hundreds of different ginsenosides have been reported; however, describing these is not within the scope of this review. For a detailed review on the structural variety of *Panax L.* species, including all ginsenosides reported up to 2012, readers should refer to (Qi et al., 2011; Yang et al., 2014). In general, based on the structure of aglycone, ginsenosides can be classified into three different types, dammarane-, oleanane-, and ocotillol-types (He et al., 2018). The dammaranne type includes 20-S-protopanaxadiols (PPD) and 20-S protopanaxatriols (PPT) which share a four-ring hydrophobic steroid-like structure with sugar moieties, but differ in the carbohydrate moieties at C3, C6, and C20. In the PPD group such as Rb1, Rb2, Rb3, Rc, Rd, Rg3, Rh2, and compound K, sugar residues are attached to the hydroxyl group at C-3 and/or C-20, while in the PPT group, such as Re, Rf, Rg1, Rg2, and Rh1, sugar moieties are attached to the hydroxyl group at C-6 and/or C-20 (**Figure 1**). Minor ginsenosides include ocotillo-type (F11) oleanane-type (Ro) ginsenosides, and other isolated compounds can be classified as modified C-20 side-chain ginsenosides (Rh4, Rg5) (Qi et al., 2011). An older classification of ginsenosides was based on their chromatographical profile, which has contributed to their current nomenclature (Ginsenoside Rb1, Rb2, Rc,…) (Yang et al., 2014).

## Interaction With Amphiphilic Molecules and Assembly into Nanostructures

Saponins are amphiphilic molecules, and can accumulate at hydrophobic/hydrophilic interfaces and self-aggregate above a certain concentration (critical micelle concentration; puriﬁed Quillaja saponins cmc (0.025wt%) (Israelachvili et al., 1977). Those properties provide the etymological background for the name “saponin” (“sapo” latin for soap), which solubilize hydrophobic molecules in aqueous solution and have similar properties to other detergents. Specific aggregation of saponins with phospholipids and cholesterol can lead to the formation of nanoparticles in aqueous solution, which can be used as carriers for drugs. Those football-shaped nanoparticles, named immunostimulating complexes (ISCOMs), can enhance the immune response toward certain antigens (Morein et al., 1987). Notably, ginsenosides have the potential to induce the formation of similar nanoparticles and could thus be used as nanocarriers for vaccines or potential anticancer drugs. More specifically, ginsenosides Ro, Rb1, and Rg1 are able to form nanoscopic aggregates in solution when combined with glucuronic acid and other saponins (Xiong et al., 2008). Ginsenoside Ro forms stable nanoscopic structures of vesicular shape. Rb1 forms worm-like and spherical micelles, whereby a higher number of sugars in the ginsenoside increases the number of binding sites between those constructs (Song et al., 2009; Dai et al., 2012; Dai et al., 2013). Red ginseng saponins extracted using an ethanol and water procedure from red ginseng roots (Wang et al., 1979) have been shown to build nanoscopic aggregates or ginsomes in the presence of cholesterol and phospholipids. Ginsomes can enhance immune responses in mice and are promising candidates for nanoparticle carriers in vaccines (Song et al., 2009).

## Ginsenoside-Membrane Interactions

### Effect of Ginsenoside Structure on Membrane Binding Property

Ginsenosides, as well as phospholipids, can be characterized as amphiphilic self-aggregating molecules. The amphiphilic character of ginsenosides permits their adsorption or insertion into lipid bilayers, in which the hydrophilic sugar moiety of saponins interacts with the interfacial part of the membrane. The membrane interface contains mainly polar headgroups, comprising osidic residues from numerous glycolipids and glycoproteins, with which the osidic part of the saponin can form intramolecular hydrogen bonds. Conversely, the steroid or triterpenoid part interacts with the membrane hydrophobic core (Keukens et al., 1995). The importance of the sugar moiety for the membrane-associated effects is highlighted by the fact that the binding of protopanaxadiol and protopanaxatriol type ginsenosides to liposomes depends inversely on the amount of sugar residues. This suggests that the higher the number of polar residues, the lower the level of binding (Hou et al., 2013). Moreover, Fukuda et al. reported that ginsenoside-Rc, having an α-L-arabinofuranose residue, exhibits remarkable agglutinability toward egg yolk phosphatidylcholine vesicles (Fukuda et al., 1985). Conversely, other saponins (Rb1, Rb2, Rd, Re, and Rg2) lack this characteristic sugar residue and present less or no agglutinability. Interaction was also found to depend on phospholipid headgroups as ginsenoside Rc interacted strongly with egg phosphatidylcholine vesicles but only slightly with egg phosphatidylethanolamine, egg phosphatidic acid, phosphatidylserine, and sphingomyelin from bovine brain. Regarding the polar sugar residues, the amount, position, type, polarity, and three-dimensional organization are important for membrane binding (Keukens et al., 1995; Hou et al., 2013; Lorent et al., 2013; Lorent J. et al., 2014).

Besides the sugar residues, the hydrophobic part of saponins is important for their binding and permeabilizing activity (Lorent J. et al., 2014; Korchowiec et al., 2015; Sudji et al., 2015), and is usually attributed to their interactions with membrane sterols. Protopanaxadiol ginsenoside-Rc interacted more efficiently with membranes composed of short or unsaturated fatty acyl chains than with those composed of saturated fatty acids (Fukuda et al., 1985). Moreover, we recently showed that cholesterol, in contrast to sphingomyelin, delays the cytotoxicity of Rh2 in human monocytic leukemia U937 cells (Verstraeten et al., 2018). This observation has been supported by a large panel of biophysical approaches on lipid monolayers or Large Unilamellar Vesicles (LUVs) (Verstraeten et al., 2019). In summary, ginsenoside membrane binding depends largely on the sugar residues, but also on the aglycone, membrane phospholipid headgroups, the unsaturation of acyl chains, and sterol content.

### Effect on Dynamic Membrane Properties

**Figure 2:**
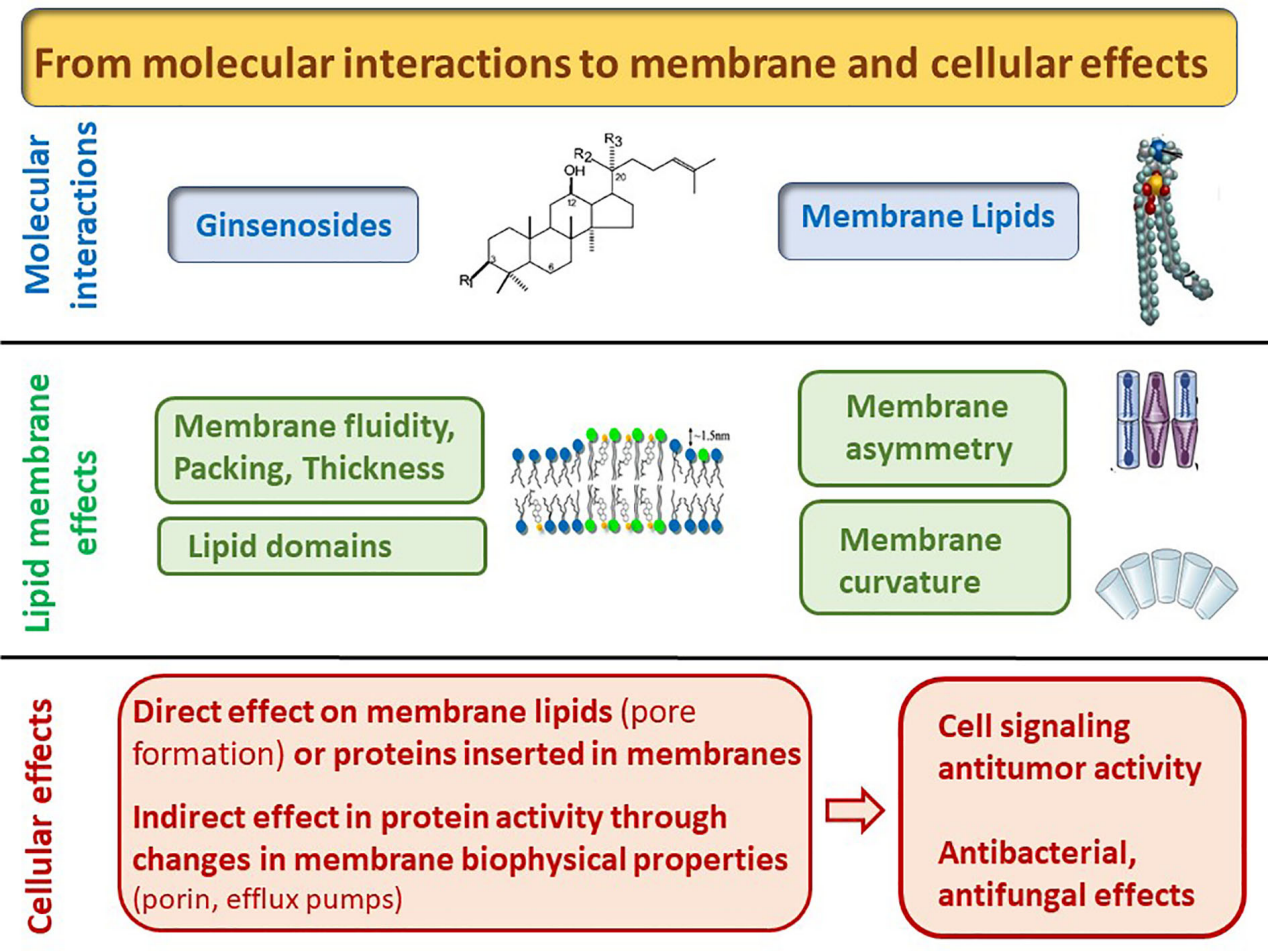
From molecular to cellular and biological effects induced by the interaction of ginsenosides with lipids.

In addition to structural considerations relating to the interaction between ginsenosides and membrane components, membranes can be defined by their collective and dynamic properties (**Figure 2**), which ultimately determine the diffusive properties of all membrane components, including exogenous ginsenoside-like substances. Membranes with a high molar ratio of cholesterol, saturated phospholipids, and sphingomyelin are tightly packed and thereby restrict lipid and protein diffusion (Barenholz, 2004; Leonard et al., 2018). Conversely, membranes with a high percentage of unsaturated phospholipids and a low cholesterol content are disordered, which enables the faster diffusion of intrinsic molecules (Filippov et al., 2007). Several signaling pathways depend on the packing of lipid membranes; therefore, a transient change in lipid membrane dynamics induced by xenobiotics can have substantial consequences on cellular physiology. Several saponins have a large influence on membrane dynamics and, thus, signaling (Lorent J. H. et al., 2014). A broad variety of protopanaxadiol and protopanaxatriol-type ginsenosides (Rb2, Rc, Rd Re, Rf, Rg1, Rg2, and Rh2) can reduce membrane packing at the interface of HeLa cell membranes (Yi et al., 2009). The aglycone protopanaxadiol can also decrease membrane packing; hence, this activity is not related to the sugar moieties (Yi et al., 2009). Insertion into the lipid bilayer reduces the mobility of phosphatidylcholine. In melanoma cells, Rh2 decreases membrane fluidity as determined by diphenylhexatriene (DPH) fluorescence anisotropy; however, no change in fluorescence is observed with trimethylammonium diphenylhexatriene (TMA-DPH), suggesting that only the inner hydrophobic core is affected (Ota et al., 1987; Jiang et al., 2010). Ginsenoside Rg3 increases the fluorescence anisotropy of DPH and TMA-DPH in multidrug-resistant KB V20 cells but not in the parental KB cell, indicating a selective effect on cancer cell membranes (Kwon et al., 2008). In bovine adrenal chromaffin cells, Rg3 increases the fluorescence anisotropy of DPH, leading to the inhibition of Na^+^ and Ca2^+^ channel activity, suggesting relationship between membrane properties and signaling (Tachikawa et al., 2001). Ginsenoside Re increases fluidity of mitochondrial membrane of brain cells after cerebral ischemia injuries in rats (DPH anisotropy) (Zhou et al., 2006). In addition, an extract of Korean red ginseng could markedly reduce DPH fluorescence anisotropy in *Candida albicans*, suggesting that its antifungal activity is related to membrane activity (Sung and Lee, 2008a; Sung and Lee, 2008b). Finally, we recently showed that Rh2 (60 µM) compacts the hydrophobic core of the lipid bilayer (DPH anisotropy) and relaxes the interfacial packaging of the phospholipid polar head (TMA-DPH anisotropy) in U937 cells (Verstraeten et al., 2018). Accordingly, by measuring the generalized polarization (GPex) of Laurdan and the anisotropy of DPH, we observed that liposomal membrane fluidity is decreased at a low ginsenoside Rh2/lipid ratio (0.4) in the presence of egg sphingomyelin and the absence of cholesterol (Verstraeten et al., 2018). Finally, the effects on membrane packing reflect the efficient insertion and interaction of ginsenosides with cellular membranes, which seem to depend largely on the cell type and lipid composition.

### Effect on Membrane Lateral Organization

In addition to the average lipid packing of headgroups and the hydrophobic core, cellular membranes display lateral heterogeneity in those parameters, reflected by lateral domains (Sezgin et al., 2017). Lipid rafts are cholesterol- and sphingolipid-enriched membrane nanodomains that facilitate protein signaling *via* the recruitment of specific proteins (Lorent et al., 2017). Their disruption may impact several signaling pathways or protein transport (Yi et al., 2009; Diaz-Rohrer et al., 2014). Ginsenosides appear to interfere with the formation of rafts, changing their properties. For example, Rp1 modulates lipid raft formation and inactivates the drug efflux pump P-glycoprotein (P-gp), leading to the accumulation of doxorubicin in doxorubicin-resistant cells (Yun et al., 2013). After 6 h, Rg3 (50 µM) has been found to reduce Aβ levels in cultured primary neurons and in the brains of mice models of Alzheimer’s disease by decreasing the association of presenilin-1 (PS1) with lipid rafts and inhibiting Υ-secretase activity (Kang et al., 2013). The effect on cultured primary neurons was already observed at 10 µM. Rh2 can also prevent Alzheimer’s disease symptoms by promoting nonamyloidogenic cleavage of amyloid precursor protein (APP) *via* a cholesterol and lipid raft-dependent pathway (Qiu et al., 2014). In addition, Rh2 has been reported to disrupt lipid rafts leading to apoptosis, either by oligomerization of the FAS death receptor in human cervical cancer HeLa cells (Yi et al., 2009) or by inactivation of the serine/threonine kinase Akt in human epidermoid carcinoma A431 and breast cancer MBA-MB-231 cells (Yi et al., 2009; Park et al., 2010). In contrast, another study showed that membrane cholesterol depletion suppresses Rh2-dependent dendrite formation in melanoma cells, without affecting lipid rafts (Jiang et al., 2010). Interestingly, results suggest that ginsenoside activity is cell-type specific. Indeed, Akt activity was decreased in lipid rafts of the glioma cell line U87 MG but increased in neuroblastoma N2a cells by 20S-protopanaxadiol through the regulation of raft-associated dephosphorylation. As 20S-protopanaxadiol has a chemical structure similar to that of cholesterol, this ginsenoside may intercalate into lipid rafts, leading to changes in the membrane microenvironment (Liu et al., 2011). The raft-disrupting ability of protopanaxadiol has been proposed to induce the activation of neutral sphingomyelinase and the successive transformation of sphingomyelin into pro-apoptotic ceramide (Park et al., 2013).

### Pore Formation

In general, pore formation by saponins depends on the interaction with cholesterol and phospholipids, and the resulting three-dimensional aggregates that form in the membrane (Keukens et al., 1995; Lorent et al., 2013). However, cholesterol is not necessary for protopanaxadiol Rb1-induced liposomal membrane permeability but suppresses its activity, whereas protopanaxatriol Rg1 does not induce membrane permeability (Yu et al., 1985). This differs from observations with other saponins, whose pore forming abilities rely on the presence of membrane cholesterol (Bangham et al., 1962; Lorent et al., 2013).

Ginsenosides Rh2 and Rg3 are effective at forming pores in red blood cells, and thereby inducing hemolysis. Conversely, numerous ginsenosides (Rc>Rd>Re - Rb1>Rg1 Rh1>Rb3 - Rg2 - R1 - F11 - PPT) can protect erythrocytes against heme-induced hemolysis (Li and Liu, 2008). These results indicate that a sugar moiety at C-20 and a hydroxyl group at C-3 play key roles in the protective effects of protopanaxadiol and protopanaxatriol, respectively (Li and Liu, 2008). Hemolytic activity does not seem to be a common feature among ginsenosides, which makes them interesting pharmacophores because of their reduced toxicity compared to other saponins. Using calcein-filled liposomes, we found that ginsenoside Rh2 induces membrane permeability in LUV containing egg sphingomyelin, and that this is reduced by cholesterol (Verstraeten et al., 2019). This result appears controversial, given that red blood cell membranes are particularly enriched in cholesterol (Wolkers et al., 2002; Deleu et al., 2014). However, we do not exclude the influence of membrane organization or the importance of other lipids, such as phosphatidylcholine in the membrane activity of Rh2. In addition, it should be noted that liposomes have some limitations, as they do not capture the full complexity of biological membranes, such as membrane asymmetry and the large number of diverse membrane proteins. The effects of an extract of red ginseng root (*Panax ginseng* C.A. Meyer) (Asian or Korean ginseng) on *C. albicans* also seem to be related to pore formation in the cellular membrane (Sung and Lee, 2008a).

### Direct Interaction With Membrane Proteins

Ginsenosides can interact with the plasma membrane, changing its dynamic and diverse cell signaling. If lipids can be considered as the main target, proteins embedded into the membrane, such as voltage-dependent or ligand-gated ion channels or pumps including the P-gp efflux pump (Nah, 2014) can also be affected directly or through an indirect effect on the biophysical membrane properties. Rg3 can interfere with the activity of ion channels, including human Kv1.4 channel and 5-hydroxytryptamine receptor type 3 (5-HT3A) (Lee et al., 2004). Rg3 inhibits 5-HT3A receptor channel activity by interacting with residues V291, F292, and I295 in the channel-gating region of transmembrane domain 2 (Lee et al., 2007; Lee et al., 2009). Moreover, in cultured hippocampal neurons, the activation of glutamate (NMDA) receptors is attenuated by ginsenoside Rh2 and Rg3 *via* a competitive interaction with its polyamine- or glycine-binding site, respectively (Kim S. et al., 2004; Lee et al., 2006). A comparative study showed that protopanaxadiol-based (Rd, Rg3, Rh2, F2, and compound K), but not protopanaxatriol-based (Rh1, F1, and Rg1) ginsenosides inhibit the sodium-glucose cotransporter (SGLT1). This suggested that the sugar moieties attached to the C-6 position of the dammarane structure may interfere in the affinity for this cotransporter (Gao et al., 2017). Consistent with this, Chen et al. (2009) showed that ginsenosides with sugar moieties attached to the C-3 position (such as PPD, Rh2, and Rg3) inhibit membrane-bound Na^+^/K^+^ ATPase activity (Chen et al., 2009). However, this inhibition is reduced or abolished when a monosaccharide is linked to the C-6 or C-20 position of ginsenoside (Chen et al., 2009).

### Membrane Antioxidant Effects

Plasma membranes have an asymmetric lipid distribution, whereby the content of unsaturated acyl chains is higher in the inner leaflet compared with the outer leaflet (Verkleij et al., 1973; Li et al., 2016). This reduces membrane packing and increases the efficiency of protein insertion (Lorent et al., 2017). However, under high oxidative stress, reactive oxygen species (ROS) can oxidize polyunsaturated lipids. This can subsequently induce a radical chain reaction leading to protein oxidation, cellular damage, and signal induction, which can promote the development of cancer, atherosclerosis, and neurodegenerative diseases (Sultana et al., 2013; Tanaka et al., 2017). Some types of ginsenosides have demonstrated a high potential for antioxidant activity and since they efficiently insert into membranes, it is reasonable to assume that their antioxidant activity targets cellular membranes. This is supported by the finding that many ginsenosides exhibit antioxidative properties, which are mainly related to the suppression of lipid peroxidation products, especially in the context of brain and myocardial injury (Zhou et al., 2006; Tian et al., 2009; Ye et al., 2011a). In a rat model of focal cerebral ischemia, Rd at concentration ranging from 0.1 to 200 mg/kg given in intraperitoneally 30 min before middle cerebral artery occlusion was found to attenuate lipid peroxidation and neuronal oxidative damage by decreasing the formation of major end-products of oxidation of polyunsaturated fatty acids (Ye et al., 2011b). Rb1 and Rg1 exhibited neuroprotective effects and prevented cardiac ischemia *via* the reduction of hydrogen peroxide and hydroxyl radicals (Deng and Zhang, 1991). Regarding the structure antioxidant-activity relationship of ginsenosides, sugar moieties at position C-20 or C-6 exert antioxidant activity. The order of antioxidative ability is as follows: Rc > Rb1 and Re > Rd > R1 > Rg1 > Rb3 > Rh1 (Liu et al., 2003). Conversely, the absence of sugar moieties at the C-20 position leads to pro-oxidant activity. This may be associated with the observation that ginsenosides 24 h incubation of Jurkat cells or colorectal cancer cells with µM concentrations of Rh2, Rh4, and Rg3 induce apoptosis *via* the formation of radical species and depolarization of the mitochondrial membrane potential (Xia et al., 2017; Wu et al., 2018). Those results highlight the complex behavior of ginsenosides under oxidative stress.

## Potential Use of Ginsenosides as Anticancer Agents

Total ginsenosides of Chinese ginseng (50 µg/ml for 24 h) showed capacity to induce cell cycle arrest and apoptosis on colorectal carcinoma HT-29 cells (Li et al., 2018). Studies on individual ginsenosides revealed the anticarcinogenic effects resulted from different mechanisms including anti-angiogenic effects (Yue et al., 2006; Yue et al., 2007) which lead to inhibition of tumor growth and metastasis (Nag et al., 2012). Some of these mechanisms may be related to their membrane activities, including alteration of lipid raft organization, pore formation, and modulation of ROS production. In addition, some ginsenosides inhibit drug efflux pumps, which can enhance the activity of conventional chemotherapeutic agents.

### Ginsenosides Affect Lipid Raft Organization

The cell membrane, especially lipid rafts, has continued to attract attention as a key factor involved in tumorigenesis. Lipid rafts serve as platforms for a number of cellular pathways related to cell survival, proliferation, and apoptosis, which are often altered in cancer cells (Mollinedo and Gajate, 2015). Consequently, it may be of great advantage to modulate these domains and associated-pathways by membrane active compounds. In this regard, several studies have demonstrated that ginsenosides Rg3 or Rh2 interfere with lipid rafts and induce antiproliferative and apoptotic effects in various cancer cells (Kim H. S. et al., 2004; Zhang et al., 2013; Huang et al., 2016). For example, Rh2 blocks the growth of glioblastomas through the inhibition of receptor-tyrosine kinase EGFR and PI3K/Akt signaling, which may be associated with lipid rafts (Li et al., 2014; Li et al., 2015).

### Ginsenosides Interact With ROS

The regulation of oxidative stress is essential in both tumor growth, metastasis, and responses to anticancer therapies. At low levels, ROS may contribute to tumor emergence either through signaling molecules or by promoting DNA mutation. At high levels under aberrant metabolism and signaling in cancer cells, ROS promote cell death and induce severe cellular damage. To overcome this, cancer cells possess antioxidant defense mechanisms to reduce ROS levels, even if this level remains high compared to normal cells (Gorrini et al., 2013). Thus, increasing oxidative stress through the generation of ROS may be an efficient way for targeted therapies to kill cancer cells. The mechanism underlying the anticancer activities of ginsenosides mediated by ROS depend on the specific type of cancer cells involved and the ginsenoside structure. Ginsenosides with sugar moieties at position C-20 or C-6 can mediate their antioxidant action through free radical scavenging to prevent tumor initiation. Conversely, ginsenosides with no sugar moieties at the C-20 position may generate ROS to promote apoptosis during tumor progression, invasion, and metastasis. For a detailed review on the role of ROS in anticancer therapy with ginsenosides, the reader is referred to (Sodrul et al., 2018).

### Ginsenosides Inhibit Efflux Pumps

Moreover, the anticancer potential of ginsenosides may result from their ability to inhibit efflux pumps, such as P-gp. P-gp overexpression is responsible for the development of multidrug resistance, which leads to the failure of chemotherapy in patients with cancer. Several ginsenosides inhibit P-gp and thus promote the chemotherapeutic activity of drugs against cancer cells (Kim et al., 2014). Rg3 inhibits P-gp activity by decreasing membrane fluidity in human carcinoma VCR-resistant KB V20C fibroblasts (Kim et al., 2003; Kwon et al., 2008). Rp1 redistributes lipid rafts resulting in the relocalization and inhibition of P-gp (Yun et al., 2013). Finally, Rg5 interacts and reverses P-gp efflux pump activity, as demonstrated by molecular docking analyses (Feng et al., 2020).

### Ginsenosides Enhance the Activity of Conventional Chemotherapeutic Agents

The combined application of ginsenosides and conventional chemotherapeutic agents could offer a promising treatment strategy for patients with cancer. Ginsenoside Rh2 (10 µM) and cisplatin (2 mg/kg) act synergistically to enhance the inhibition of human ovarian tumor cell growth in nude mice (Kikuchi et al., 1991). In combination with the alkylating agent cyclophosphamide, ginsenosides Rg3 and Rh2 decrease tumor growth in mice (Nakhjavani et al., 2019). Beside their own anticancer activities, it is tempting to speculate that ginsenosides could improve the antitumoral activity of chemotherapeutic agents through an increased membrane permeability and decreased efflux.

### Restriction as Anticancer Agents

Currently, saponins are not used as chemotherapeutic agents due to their intrinsic hemolytic activity and poor bioavailability, resulting from their low aqueous solubility, instability in the gastrointestinal tract, and extensive metabolism in the body. Nevertheless, some saponins have shown low or no hemolytic effects, depending on their chemical structure (Namba et al., 1973). Re, Rh1, and Rh2 demonstrate adjuvant potential with low hemolytic activity (Sun et al., 2005; Sun et al., 2006; Yang et al., 2007). Numerous drug delivery systems, such as liposomes or nanostructures, have been developed to overcome the rapid plasma elimination of ginsenosides and to increase their solubility, leading to improved antiproliferative effect while preventing hemolytic activity (Kim et al., 2018). Indeed, Rh2-loaded methoxy poly(ethylene glycol)-poly(lactide) (mPEG-PLA) liposomes (Rh2-PLP) suppress tumor growth in HepG2-xenografted mice without any significant toxicity (Xu et al., 2015). Ginsenoside compound K encapsulated in phosphatidylcholine and phosphatidylethanolamine polyethylene glycol (PEG) enhances solubility and oral bioavailability compared to free compound K. This micellar system improves the antitumor effects of ginsenoside, including cell-cycle arrest, and decreases xenograft tumor growth in mice with low toxicity (Jin et al., 2018). In addition, treatment with G-Rg3 bile salt-phosphatidylcholine-based mixed micelle systems did not induce hemolysis in erythrocytes and demonstrated higher antiproliferative activity against tumor cells compared to free Rg3 (Yu et al., 2015). Finally, a new drug-delivery system based on highly porous graphene treated with ginsenoside Rh2 has been recently generated and shown to improve their anticancer activities (Zare-Zardini et al., 2018).

## Potential Use of Ginsenosides Against Invasive Microorganisms

### Fungi

Many saponins have demonstrated antifungal properties, functioning in host chemical defenses to protect plants from fungal invasion (Morrissey and Osbourn, 1999). The major mechanism underlying this effect is the formation of complexes with membrane ergosterol, the main sterol of fungi, leading to loss of membrane integrity (Keukens et al., 1995). The importance of ergosterol for saponin activity is emphasized by the isolation of insensitive mutants (*Fusarium solani*) to saponin due to their low sterol content (Defago and Kern, 1983). Fungi also counteract saponin activity by producing saponin-detoxifying enzymes. Ginseng root pathogens, *Pseudogymnoascus destructans* and *Pythium irregulare*, hydrolyze monosaccharide moieties attached to the C-3 and C-20 position of PPD-type ginsenoside by extracellular glycosidase, rendering them resistant to ginsenoside toxicity (Yousef and Bernards, 2006; Zhao et al., 2012). Consistent with this, removal of the sugar chain attached to the C-3 position has been shown to restrict the membrane binding of saponin to 3β-hydroxyl sterols (Kazan and Gardiner, 2017). The position and number of ginsenoside sugar moieties also influence the antifungal activity. Indeed, regarding the position of sugar moieties, the protopanaxatriol-type ginsenoside fraction (PPT-GF; Re, Rg1) from the roots of *Panax ginseng* C.A. Meyer (Asian or Korean ginseng) demonstrated higher growth inhibition against five ginseng non-pathogens compared to the protopanaxadiol-type ginsenoside fraction (PPD-GF; Rb2, Rc, Rd) (Zhao et al., 2012). The antifungal activity of ginsenosides is negatively correlated with the number of sugar moieties. Fungal membranes are disrupted to a greater extent after exposure to less polar ginsenosides (ginsenoside-Rk3, -Rh4, -Rh5) compared to polar ginsenosides (notoginsenodise-R1, ginsenoside Rg1, -Re, Rb2, Rd). This may be because lower polar ginsenosides interact in a larger extent with fungal membranes. Mechanistically, the interaction between ginsenosides isolated from Korean red ginseng and the *Candida albicans* membrane decreased DPH fluorescence anisotropy and disrupted the structure of the cell membrane (Sung and Lee, 2008a). Interestingly, plant cells contain phytosterols such as campesterol, β-sitosterol, and stigmasterol in their membranes, whereas fungal plant pathogens contain ergosterol. This suggests that targeting fungal sterols *via* ginsenoside synthesis may be an effective and non-toxic host defense strategy for plants to counteract fungal infections (Kazan and Gardiner, 2017).

### Bacteria

The overuse of antibiotics has led to the development of bacterial resistance, resulting in an increased interest in alternative therapies, particularly in the therapeutic use of plant products. Plants have developed multiple defense mechanisms against both fungal and bacterial invasions involving the synthesis of saponins. Ginseng has demonstrated antibacterial effects against pathogenic Gram-positive and Gram-negative bacteria (Kachur and Suntres, 2016). As noted with regards to their antifungal activity, less polar ginsenosides (-Rg2, -Rg3, -Rg6, -F4, -Rg5, and –Rk1) present higher antimicrobial activity against three bacterial species (*Fusobacterium nucleatum*, *Clostridium perfringens*, and *Porphyromonas gingivalis*), compared with polar ginsenosides (-Rg1, -Rc, Rb2, –Rd) due to their disruptive activity on bacterial membranes (Xue et al., 2017). Interestingly, the combination of Korean red ginsenosides and kanamycin improves antibacterial activity against methicillin-resistant *Staphylococcus aureus* (MRSA). The partial disruption of the bacterial membrane by ginsenosides is believed to facilitate the entry of kanamycin, and could explain these synergistic antibacterial effects (Sung and Lee, 2008b). In addition, non-toxic doses of ginsenoside Rh2 have been shown to enhance the susceptibilities of *Staphylococcus aureus* and *Escherichia coli* to ciprofloxacin both *in vitro* and *in vivo* (Kim et al., 2012). This effect of Rh2 could be attributed to the increased accumulation of ciprofloxacin *via* the inhibition of NorA efflux pump embedded in bacterial membranes (Zhang et al., 2014). Regarding this activity, ciprofloxacin-loaded polymeric micelles (poloxamer/phosphatidylcholine/cholesterol) including ginsenoside Rg3 (0.2 mg/ml) have been shown to simultaneously inhibit the P-gp efflux pump and improve ciprofloxacin solubility likely through an enhanced capacity for drug loading (Sharif Makhmal et al., 2018). Since bacterial membranes do not possess sterols, it is tempting to speculate that the antibacterial activity of ginsenosides may result from interaction with hopanoids (pentacyclic triterpenoids, structurally similar to steroids) or from other unrelated mechanisms.

## Conclusions

The plasma membrane is a key target for ginsenosides, which act *via* the modulation of essential membrane proteins and the reorganization of lipid bilayers. This review provides an overview of different studies that have investigated the physicochemical properties of different ginsenosides and their effects on membrane components embedded in both artificial and biological membranes. We highlight the diverse effects of ginsenosides, which could result from the following attributes. First, more than 100 ginsenosides have been isolated, which differ in their amphiphilic structure, conferring them specific and multiple membrane activities. Second, membrane activities of ginsenosides seem to change depending on the cell type, which could be explained by differences in the composition of the lipid membrane between cell lines. Third, by changing membrane dynamics, a single ginsenoside may alter a range of membrane proteins, thereby altering various cell signaling pathways. Interestingly, the ability of several ginsenosides to suppress cell proliferation, induce apoptosis, and inhibit efflux pumps suggests they could represent promising candidates for drug development for the treatment of cancer, as well as bacterial and fungal infections.

## Author Contributions

SV and JL collected data and wrote the manuscript with the participation from M-PM-L. All authors contributed to the article and approved the submitted version.

## Conflict of Interest

The authors declare that the research was conducted in the absence of any commercial or financial relationships that could be construed as a potential conflict of interest.
